# Convolutional neural network for detecting rib fractures on chest radiographs: a feasibility study

**DOI:** 10.1186/s12880-023-00975-x

**Published:** 2023-01-30

**Authors:** Jiangfen Wu, Nijun Liu, Xianjun Li, Qianrui Fan, Zhihao Li, Jin Shang, Fei Wang, Bowei Chen, Yuanwang Shen, Pan Cao, Zhe Liu, Miaoling Li, Jiayao Qian, Jian Yang, Qinli Sun

**Affiliations:** 1 Department of Radiology, The First Affiliated Hospital of Xi’an Jiaotong University, Yanta West Road No. 277, Xi’an, 710061 China; 2 The Key Laboratory of Biomedical Information Engineering, Ministry of Education, Department of Biomedical Engineering, School of Life Science and Technology, Xi’an Jiaotong University, Xi’an, 710054 China; 3 InferVision Institute of Research, Beijing, 100025 China; 4 Academy for Advanced Interdisciplinary Studies, Peking University, Beijing, 100191 China; 5 Department of Medical Imaging, No. 215 Hospital of Shaanxi Nuclear Industry, Xianyang, 712000 China; 6 GE Healthcare, Xi’an, 710076 China; 7 School of Information Science and Technology, Northwest University, Xi’an, 710127 China; 8 Department of Radiology, Tuberculosis Hospital of Shannxi Province (The Fifth People’s Hospital of Shaanxi Province), Xi’an, 710100 China

**Keywords:** Rib fracture, Convolutional neural network, YOLO, Detection model, Radiograph

## Abstract

**Background:**

Chest radiography is the standard investigation for identifying rib fractures. The application of artificial intelligence (AI) for detecting rib fractures on chest radiographs is limited by image quality control and multilesion screening. To our knowledge, few studies have developed and verified the performance of an AI model for detecting rib fractures by using multi-center radiographs. And existing studies using chest radiographs for multiple rib fracture detection have used more complex and slower detection algorithms, so we aimed to create a multiple rib fracture detection model by using a convolutional neural network (CNN), based on multi-center and quality-normalised chest radiographs.

**Methods:**

A total of 1080 radiographs with rib fractures were obtained and randomly divided into the training set (918 radiographs, 85%) and the testing set (162 radiographs, 15%). An object detection CNN, You Only Look Once v3 (YOLOv3), was adopted to build the detection model. Receiver operating characteristic (ROC) and free-response ROC (FROC) were used to evaluate the model’s performance. A joint testing group of 162 radiographs with rib fractures and 233 radiographs without rib fractures was used as the internal testing set. Furthermore, an additional 201 radiographs, 121 with rib fractures and 80 without rib fractures, were independently validated to compare the CNN model performance with the diagnostic efficiency of radiologists.

**Results:**

The sensitivity of the model in the training and testing sets was 92.0% and 91.1%, respectively, and the precision was 68.0% and 81.6%, respectively. FROC in the testing set showed that the sensitivity for whole-lesion detection reached 91.3% when the false-positive of each case was 0.56. In the joint testing group, the case-level accuracy, sensitivity, specificity, and area under the curve were 85.1%, 93.2%, 79.4%, and 0.92, respectively. At the fracture level and the case level in the independent validation set, the accuracy and sensitivity of the CNN model were always higher or close to radiologists’ readings.

**Conclusions:**

The CNN model, based on YOLOv3, was sensitive for detecting rib fractures on chest radiographs and showed great potential in the preliminary screening of rib fractures, which indicated that CNN can help reduce missed diagnoses and relieve radiologists’ workload. In this study, we developed and verified the performance of a novel CNN model for rib fracture detection by using radiography.

## Background

Thoracic trauma is a common injury encountered in the emergency department and accounts for approximately 10%–15% of all trauma cases [1]. The mortality rate globally ranges from 20 to 25% [2]. Traumatic rib fracture, caused by a tremendous impact force on the chest wall, is the most common form of blunt thoracic injury and accounts for approximately 35% of all cases of thoracic traumas [3]. Rib fractures are associated with significant morbidity and mortality, both of which increase as the number of fractured ribs increases [4, 5]. Hence, rib fracture is an essential indicator of trauma severity. Accurately detecting rib fractures, compared to other injuries, can result in a higher treatment rate, avoid complications, and help solve medical-legal disputes such as traffic accidents and physical fighting [6].

Chest radiography and computed tomography (CT) are two main methods for detecting rib fractures. Chest radiography is usually the initial imaging modality for rib fracture screening because the radiation dose, time and economic cost of CT are all higher than those of radiography [7–9]. The American College of Radiology criteria for the evaluation of rib fractures recommend chest radiography with a posteroanterior view at four variant evaluations for suspected rib fractures in non-high-energy blunt trauma [8]. Chest radiography is also a complementary examination for high-energy blunt trauma [10]. However, research has shown that the overall incidence of rib fractures is probably higher than that previously recognised [11]. A previous investigation reported that up to 50% of rib fractures may be missed on plain radiographs, which may lead to potential risks to patients [12]. The detection of rib fractures on chest radiographs depends mostly on the reader’s experience, quality of the displayed images, and/or clinical scenario of chest radiograph scanning. Rib fracture detection is a time-consuming and demanding task for radiologists. Thus, a fast, easily available, and highly accurate method for rib fracture screening, which could be adopted to relieve radiologists and develop a cost-effective tool for clinical application, is urgently needed.

Artificial intelligence (AI) is widely used in the medical field, particularly in radiology. The deep learning algorithm of AI demonstrates good diagnostic accuracy and can be used to improve the quality and speed of image interpretation and increase the efficiency of physicians [13–15]. Convolutional neural network (CNN) is an essential branch of deep learning. The multiple processing layers of CNN are more sensitive to image features and can enhance recognition accuracy [16], which are commonly used AI techniques in medical imaging among radiology researchers [17, 18]. Yamashita et al. [16], divided the application of CNN into classification, segmentation, detection, and other applications [16] such as lung nodule classification [19], liver segmentation [20] and breast cancer detection [21]. CNN also demonstrates high feasibility and potential for fracture detection. Studies on lateral wrist fractures, proximal humerus fractures, thighbone fractures and orthopaedic trauma have shown promising results [22–25].You Only Look Once v3 (YOLOv3) is a classic CNN algorithm with an excellent network structure, which is performed well in objection detection but seldom been used in the rib fractures detection.

For rib fracture detection, several research studies have been conducted, based on CNN. Jin et al. [26], developed an automatic system, named FracNet, which is based on 3D UNet, to detect and segment rib fractures on CT images and achieved a detection sensitivity of 92.9% [26]. Weikert et al. [27] constructed a deep learning-based algorithm to detect acute and chronic rib fractures on trauma CT images, and it achieved good performance with a sensitivity of 87.4% [27]. Yang et al. [28] verified that the use of a deep learning system could be used to diagnose and classify rib fractures with better efficiency, faster speed, and similar results as those of radiologists’ readings [28]. However, previous work has primarily focused on CT images, but few studies have verified the performance of the CNN model for detecting rib fractures by using radiography. Compared to CT, radiography is usually the first choice for diagnosing rib fractures in a clinical environment. Some object detection algorithms have been tested to detect fractures, based on radiography such as Faster RCNN [29], Libra RCNN [30], Dynamic RCNN [31], Cascade RCNN [32] and CCE-Net [33]. While these methods were all be tested in single center dataset and without the application of YOLOv3. In this study, we applied YOLOv3 because YOLOv3 is a faster, relatively easier to comply, and more convenient framework than the CNN frameworks mentioned before [34]. YOLOv3 has also been proven to have very good performance in multi-target detection. The traditional single detection method is more prone to miss detection for rib fractures because multiple rib fractures are more common for patients with rib fractures, instead single rib fractures are less common, so the multiple rib fracture detection model is very meaningful.

Thus, the aim of this study was to create a novel model for multiple rib fracture detection by using a CNN, based on multicenter and quality-normalised chest radiographs. The contributions of our study can be listed as follows. First, radiographs from four hospitals and external validation were collected, and the multicenter dataset could improve the robustness of the model. Second, image quality normalisation using the multiscale image contrast amplification (MUSICA) algorithm has been chosen, which is excellent in image enhancement and could ensure the model’s consistency but seldom applied in radiographs [35]. Third, A CNN model was then constructed using the YOLOv3 algorithm, which is seldom used to detect rib fractures and achieved an outstanding detection rate in our study. Finally, the detection ability of the CNN model was compared with that of junior and senior radiologists and found the performance is better than the junior radiologists and similar to the senior radiologists. This study is the first to develop and verify the performance of a CNN model for detecting rib fractures on chest radiographs through normalised images from a multicenter dataset.

## Patients and methods

### Study design and patients

**Fig. 1:**
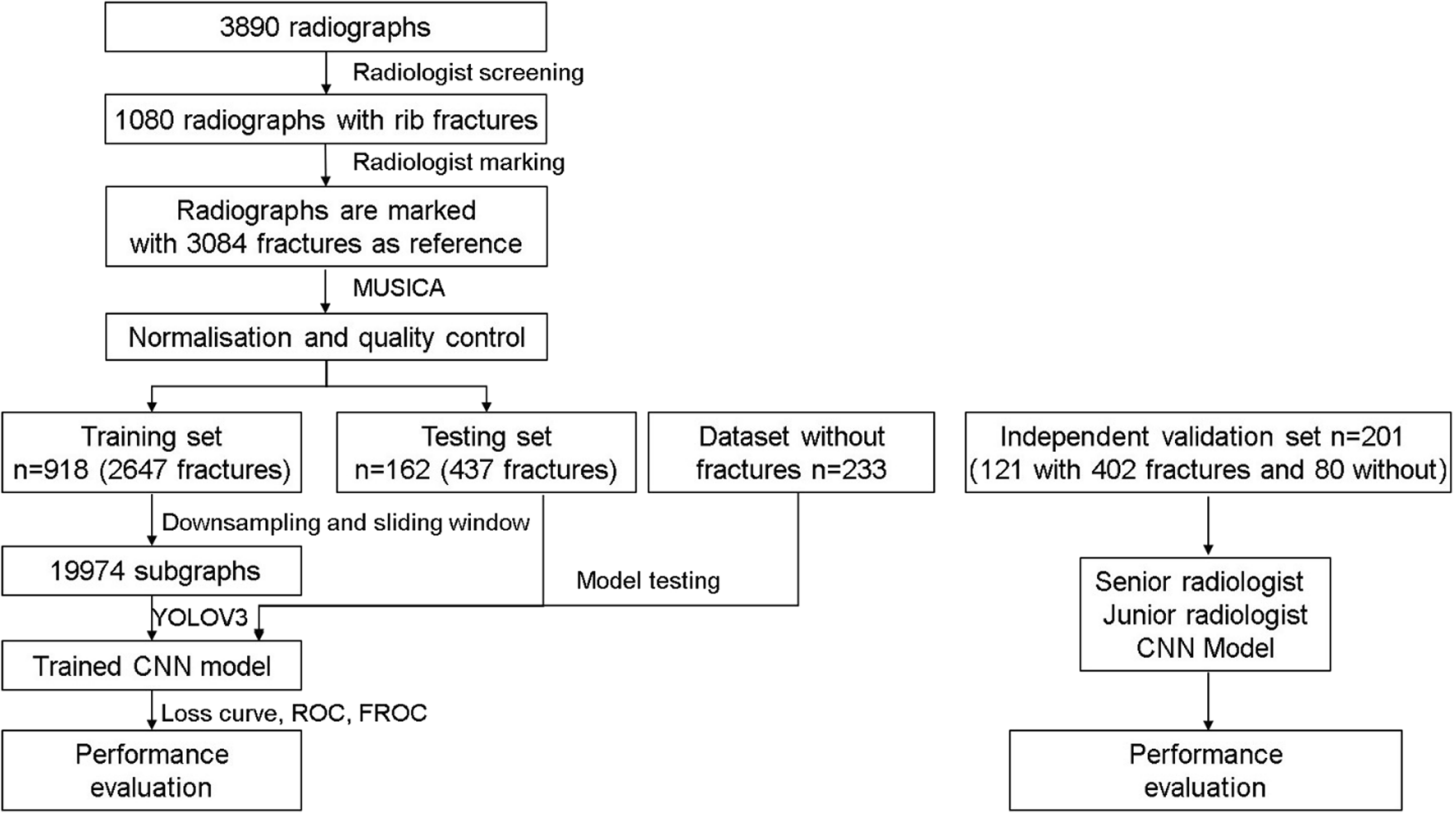
The study workflow includes radiograph screening, graph preprocessing, model training and evaluation. *MUSICA* multiscale image contrast amplification, *CNN* convolutional neural network, *ROC* receiver operating characteristic, *FROC* free-response receiver operating characteristic

This retrospective study used only anonymised data. All chest radiographs were obtained from four local hospitals between 9 July 2017 and 25 June 2019 and were all adult aged from 18 to 70. The scanners included Ysio and AXIOM Aristos FX (both: SIEMENS, Munich, Germany), Definium 6000 digital radiography (DR) (GE Healthcare, Chicago, IL, USA), DigitalDiagnost VS (Philips Healthcare, Amsterdam, Netherlands), Carestream CARESTR DR (Philips Healthcare), N600 DR (Neusoft Medical Systems, Shenyang, China), and D50S DR system (Toshiba, Tokyo, Japan). All images were stored in the Digital Imaging and Communications in Medicine format and reviewed by radiologists using InferScholar (https://research.infervision.com; Beijing, CHINA). The graph size varied from 1576 × 1960 to 3072 × 3072. A total of 3890 chest radiographs (one patient per one image) from patients aged 18–70 years were analysed for preliminary screening. Three radiologists with more than 15 years of radiological experience independently interpreted the images with relevant clinical information (e.g. palpation results and clinical history). Radiographs with the indication of no rib fracture, postoperative internal fixation of the rib, poor quality breathing, and surface foreign bodies that affected the diagnosis were excluded. Inconsistencies were resolved through discussion. In total, 1080 radiographs with rib fractures were obtained. The work diagram is illustrated in Fig. 1.

**Fig. 2:**
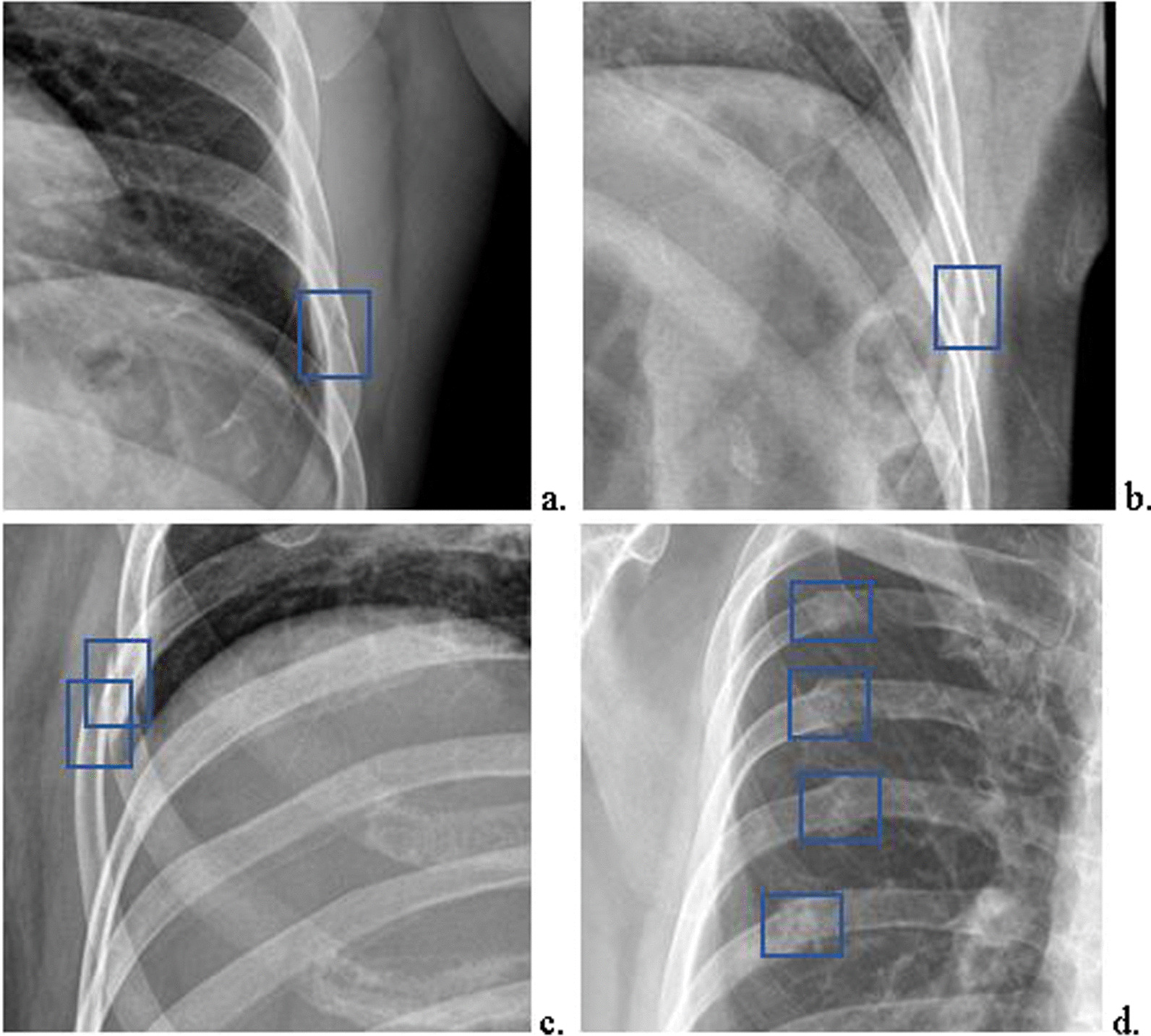
Fracture sites in 1080 radiographs with the following signs were marked by the radiologists. **a** Complete rip disruption with a lucent line. **b** Disruption of the inner or outer cortex. **c** Fracture rib end displaced. **d** Rib deformity with callus formation

To collect data for constructing the CNN-based rib fracture detection model, the radiologists marked the fractures on the graphs. One radiologist marked the fracture sites on 1080 radiographs with the following signs: (1) complete rip disruption with a lucent line, (2) disruption of the inner or outer cortex, (3) fracture rib end displacement and (4) rib deformity with callus formation. To reduce the mark error, another radiologist confirmed all markers. As shown in Fig. 2, the training set contains 912 of the 1080 radiographs containing rib fractures, and the testing set contains 168 of the 1080 radiographs containing rib fractures and 233 radiographs not containing rib fractures. An additional validation set contains 201 chest radiographs collected from different period, 121 of which have rib fractures and 80 of which have not, and the validation set was confirmed by the same three radiologists with more than 15 years of radiological experience. One junior radiologist with 5 years of experience and one senior radiologist with 10 years of experience were also recruited for the rib fracture reading experiment.

### Data processing

#### Image quality improvement using MUSICA

**Fig. 3:**
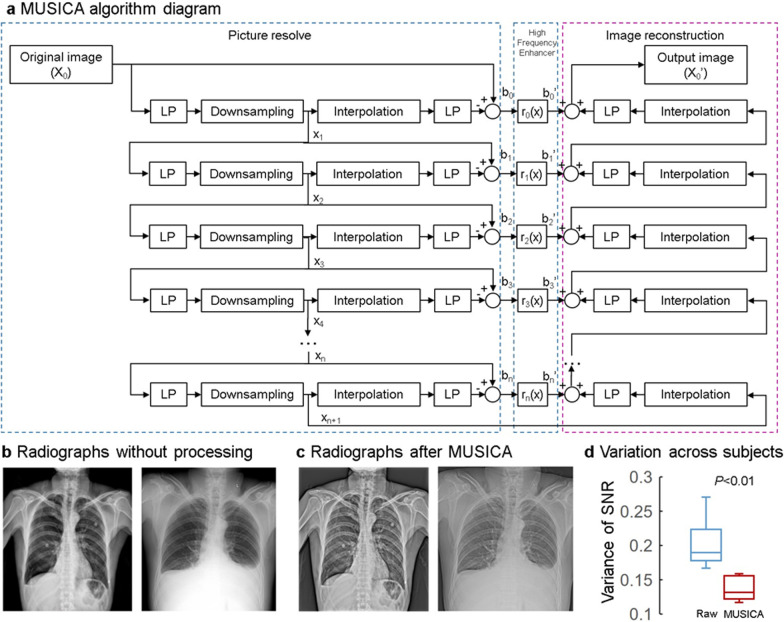
Radiograph preprocessing, based on the multiscale image contrast amplification (MUSICA) algorithm. **a** The MUSICA algorithm diagram with picture resolve, high-frequency enhancer, and image reconstruction. **b** Two radiographs from different machines. **c** The corresponding graphs processed by MUSICA. Compared to the raw data, the processed graphs have a more consistent image quality, especially the rib display. **d** Variance of the signal-to-noise ratio (SNR) of the images before and after MUSICA processing

Owing to the diversity of data sources, the use of MUSICA is inevitable before CNN model training to reduce data heterogeneity from the four different hospitals with different imaging qualities. MUSICA [36] involves the following three steps (Fig. 3): (1) Gaussian pyramid decomposition of the image, (2) enhancement of the high-frequency (detailed) part of the image, and (3) image reconstruction.

#### Architecture of the CNN

**Fig. 4:**
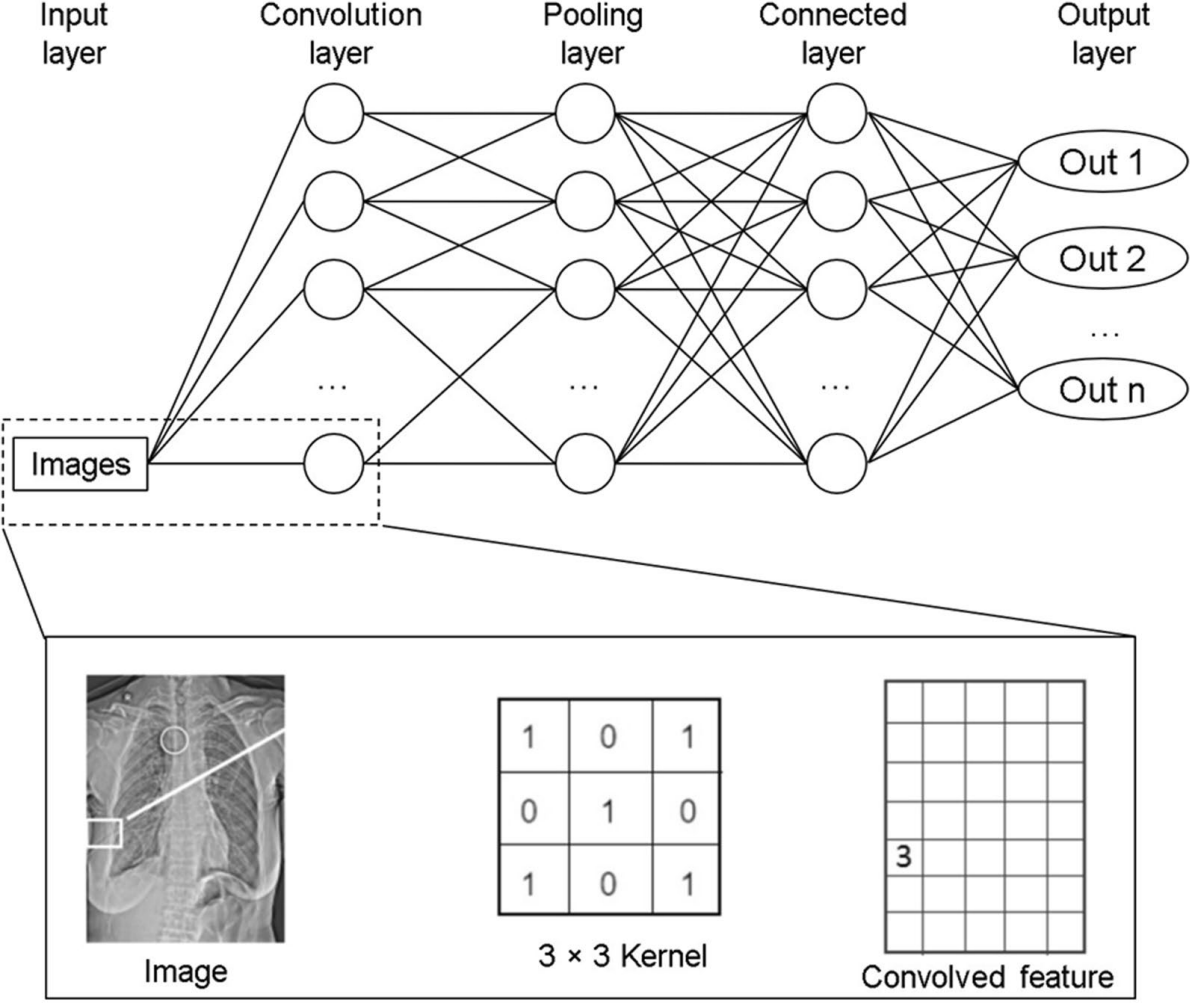
Convolutional neural network (CNN) architecture and operations used in this study. The white box picks a matrix of pixel value and then convolutes with a kernel to obtain the feature matrix

A CNN is formed by stacking the input, convolution, pooling, fully connected, and output layers. The input layer is the first layer of a CNN, and the input to a CNN consists of raw images, which are vectors in two or three dimensions. The convolutional layer, which is the core of a CNN, generally consists of a set of learnable filters or kernels with small perceptual fields. Each convolutional kernel has parameters such as the kernel size, padding, and stride. The inner product operation is performed sequentially from the top-left corner of the image to extract the high-level features of the image. The pooling layers do not change the depth of the network; however, they can downsize the matrices and reduce the number of nodes in the last fully connected layer to reduce the risk of overfitting. After several rounds of convolution and pooling layer processing, one or two fully connected layers are at the end of the neural network to generate the results. For classification tasks, a higher number of layers represents amplified input aspects, which are crucial for discrimination and suppressing irrelevant variations (Fig. 4).

#### Establishment of the training and testing sets

**Fig. 5:**
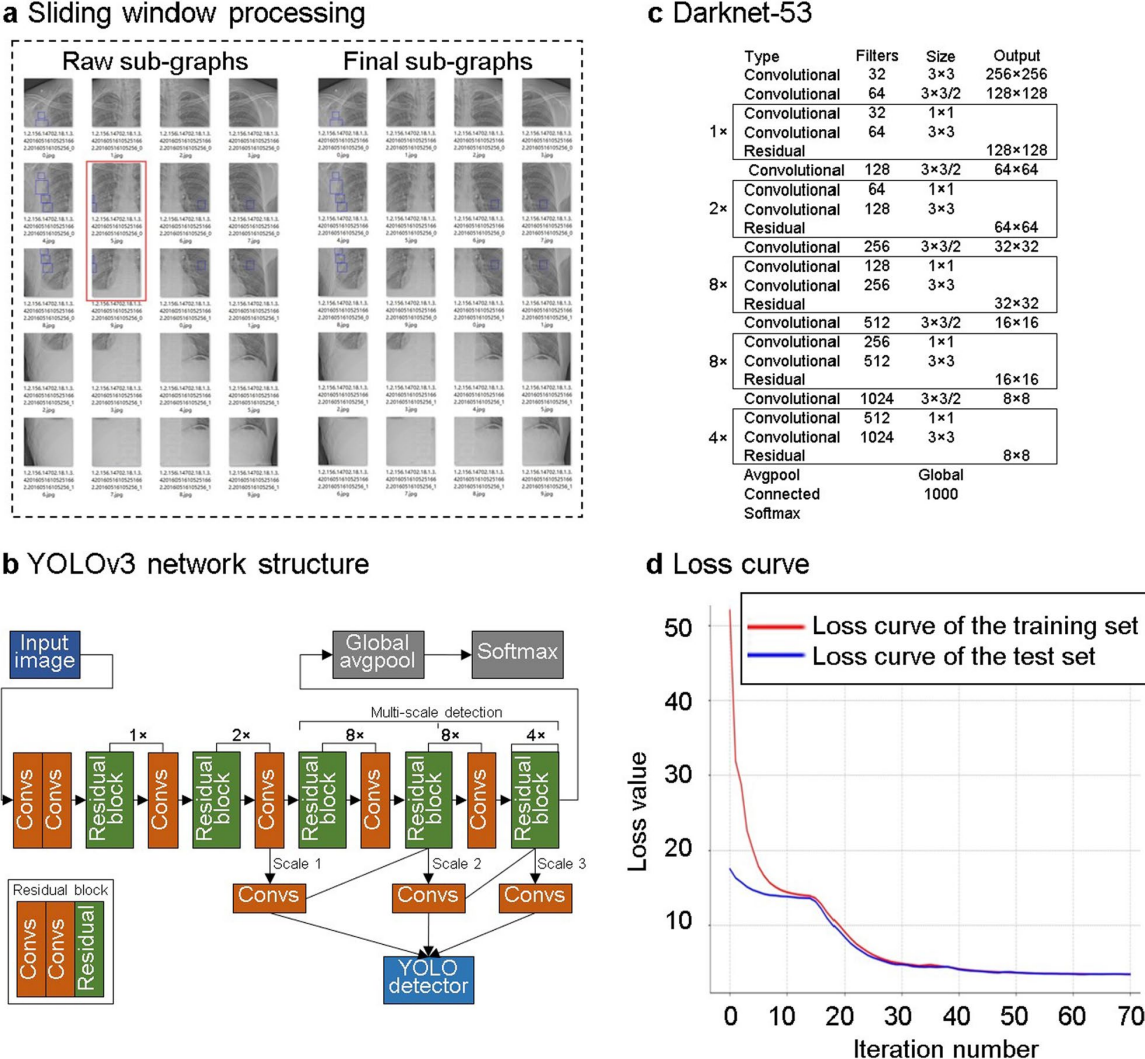
Model training workflow. **a** Sliding window processing. **b** YOLOv3 network structure. **c** Darknet-53. **d** Loss curves. YOLOv3, You Only Look Once, version3 algorithm

The 1080 images after MUSICA were randomised into the training set and the testing set with 918 graphs and 162 graphs, respectively. An additional 233 radiographs without rib fractures were also added to the testing set as the joint testing group to evaluate model generalisability. In previous studies, radiographs were manually cropped into a square or to centre the objective [23]. The current study incorporated two additional steps for segmenting the radiographs and amplifying the data. First, the input image was downscaled from 2458 × 2881 to 1229 × 1440. Second, a sliding window was used to generate subgraphs with a window size of 512 × 512 in steps of 256. Each image was divided into approximately 20 subgraphs (Fig. 5a). Once the marked area was cut, the smaller area was filtered out, and the training data were generated after filtering (Fig. 5a). With regard to the training set, 19,974 subgraphs were generated by the sliding window and sent to a deep learning network for training and testing.

#### Network training

YOLOv3 (https://pjreddie.com/darknet/yolo/) is a classic CNN algorithm with an excellent network structure (Fig. 5b). This model has several inherent advantages: fast evaluation, multiscale predictions, and a better backbone classifier. First, Darknet-53 was trained as the backbone for object detection. Darknet-53 (Fig. 5c) consists of 53 convolutional layers and one fully connected layer. Several consecutive 1 × 1 and 3 × 3 convolutions were added, and the first 52 layers were used to extract the image features. The k-means algorithm was used to count the size of the fracture marker in the labelled sample. To better detect the location of the fracture, each cell was responsible for predicting four anchors. One of these cells was selected as the prediction result, which used a total of 12 anchors: (54, 58), (61, 76), (65, 59), (69, 99), (74, 71), (80, 60), (85, 84), (94, 116), (104, 69), (111, 91), (122, 209) and (139, 123). Each box was classified using logistic regression analysis to determine whether the fracture area was included. After 50 iterations of network training, the losses of the training and testing sets were no longer reduced, indicating that the network converged to a stable state, as shown by the loss curve in Fig. 5d.

### Statistical analysis

The chi-square test was used to compare the performance of the CNN model with that of the senior radiologist and junior radiologist. To evaluate model performance, a conventional receiver operating characteristic (ROC) analysis was performed to examine model sensitivity and false-positive results. Conventional ROC analysis was also used to explore the model’s ability in the joint testing set (162 radiographs with rib fractures and 233 radiographs without rib fractures). The multilesion detection rate was assigned to the model by using the free-response ROC (FROC) in the testing set. FROC defines the lateral axis as the overall average of false detections and the vertical axis as the true positive. Accuracy, area under the curve (AUC), sensitivity/specificity, and 95% confidence intervals (CI) were determined. All statistical analyses were performed using Python script (https://www.python.org).

## Results

### MUSICA pre-processing performance

After MUSICA, the contrast uniformity between the bone and lung tissues was significantly improved (Fig. 3). Although the raw images behaved differently with considerable differentiation of contrast and detail, the two processed images appeared to be similar in image quality and contrast.

### Deep learning YOLOv3 network performance

**Fig. 6:**
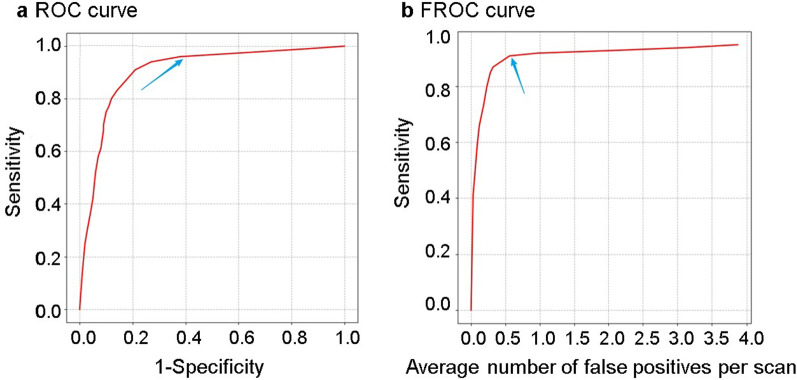
Performance of the testing model. **a** The receiver operating characteristic (ROC) curve and **b** the free-response ROC (FROC) curve

The training set included 918 patients with 2647 fractures. The CNN model detected 3580 fractures, of which 2435 were detected correctly, 212 were missed and 1145 were false positives. The test set included 162 patients with 437 fractures. The model detected 488 fractures, of which 398 were detected correctly, 39 were missed, and 90 were mistakenly detected. In the training set, the sensitivity (i.e. the number of fractures detected correctly divided by the number of marked fractures) was 92.0%, and the precision (i.e. the number of fractures detected correctly divided by the number of fractures detected) was 68.0%. In the test set, the sensitivity was 91.1% (Table 1). In the testing set, the multilesion detection rate was also verified with FROC. When the false-positive rate was set as 0.56, the sensitivity of the whole lesion detection reached 91.3% (Fig. 6b).

**Table 1:** Sensitivity and precision of the CNN model in the training and testing sets

Data	Marked fractures	Detected fractures	Correctly detected fractures	Sensitivity (%)	Precision (%)
Training set	2647	3580	2435	92.0	68.0
Testing set	437	488	398	91.1	81.6

Sensitivity is number of fractures detected correctly divided by the number of marked fractures. Precision is the number of fractures detected correctly divided by the number of fractures detected

*CNN* convolutional neural network

Radiographs without rib fractures were added to the test set to evaluate the ability of the model to detect rib fractures. Finally, 395 radiographs with 162 fractures and 233 without fractures were included in the study. The CNN model detected 199 radiographs with fractures and 196 radiographs without fractures. The accuracy was up to 85.1%, and the sensitivity and specificity were 93.2% and 79.4%, respectively (Table 2). ROC analysis showed that the AUC reached 0.92 (95% CI 0.86–0.96) (Fig. 6a).

**Table 2:** Detection rate of the CNN model in the testing set, based on case level

CNN model	Chest radiograph		Total
	With rib fractures	Without rib fractures	
Detected fractures	151	48	199
Undetected fractures	11	185	196
Total	162	233	395

Sensitivity is 93.2% [TP/(TP + FN) × 100% = 151/162 × 100%]. Specificity is 79.4% [TN/(TN + FP) × 100% = 185/233 × 100%]. The positive predictive value (PPV) is 75.9% [TP/(TP + FP) × 100% = 151/199 × 100%]. The negative predictive value (NPV) is 94.4% [TN/(TN + FN) × 100% = 185/196 × 100%]. Accuracy is 85.1% [(TP + FN)/(TP + FN + TN + FN) × 100% = (151 + 185)/395 × 100%]

*CNN* convolutional neural network, *TP* true positive, *FN* false negative, *TN* true negative, *FP* false positive

### Reading experiment

With regard to the experimental results at the fracture level, the CNN model detected 97 radiographs with 437 fractures, of which 351 were detected correctly, 51 were missed and 86 were false positives. The senior radiologist recognised 125 radiographs with 392 fractures, of which 323 were correctly detected, 79 were missed, and 69 were false positives. The junior radiologist identified 130 radiographs with 361 fractures, of which 295 were correct, 107 were missed, and 66 were false. The sensitivity and precision of the detection by the CNN model, senior radiologist, and junior radiologist were 87.3%, 80.3%, and 80.3%, respectively, and 82.4%, 73.4%, and 81.7%, respectively. The sensitivity of detection was significantly higher in the CNN model than among the junior radiologist (P = 0.01), which indicated that the CNN model had better detection ability. Meanwhile, no significant difference existed between the senior and junior radiologists or between the CNN and senior radiologist (P > 0.05) (Table 3).

**Table 3:** Comparison of sensitivity and precision in the independent testing group, based on fracture level

Data	Marked fractures	Detected fractures	Correctly detected fractures	Sensitivity	Precision
CNN model	402	437	351	87.3%	80.32%
Senior radiologist	402	392	323	80.3%	82.40%
Junior radiologist	402	361	295	73.4%	81.72%
*P*_1_	NA	NA	NA	0.15	0.57
*P*_2_	NA	NA	NA	0.13	0.43
*P*_3_	NA	NA	NA	0.01	0.43

Sensitivity is the number of fractures detected correctly divided by the number of fractures marked. Precision is the number of fractures detected correctly divided by the number of fractures detected. *P*_1_ is the P value for the senior radiologists versus the junior radiologists. *P*_2_ is the P value for the CNN versus the senior radiologist. *P*_3_ is the P value for the CNN versus the junior radiologist. Comparisons were conducted by using the chi-squared test

*NA* not available, *CNN* convolutional neural network

For the model’s detection ability at the case level, the CNN model detected 130 radiographs with fractures and 71 without fractures. The senior radiologist identified 125 fractures and 76 without fractures. The junior radiologist identified 97 fractures and 104 without fractures. The accuracy and sensitivity of the identification by the CNN model, senior radiologist, and junior radiologist were 91.5%, 96.7%, 94.0%, respectively, and 96.7%, 85.1%, and 77.7%, respectively (Table 4).

**Table 4:** Detection rate of marked fractures in the independent testing set at the case level

CNN model	Chest radiograph		Total
	With rib fractures	Without rib fractures	
*(a) CNN model*			
Detected fractures	117	13	130
Undetected fractures	4	67	71
Total	121	80	201

Senior radiologist	Chest radiographs		Total
	With rib fractures	Without rib fractures	
*(b) Senior radiologist*			
Detected fractures	117	8	125
Undetected fractures	4	72	76
Total	121	80	201

Junior radiologist	Chest radiographs		Total
	With rib fractures	Without rib fractures	
*(c) Junior radiologist*			
Detected fractures	94	3	97
Undetected fractures	27	77	104
Total	121	80	201

Model	Sensitivity (%)	Specificity (%)	PPV (%)	NPV (%)	Accuracy (%)
*(d) Sensitivity, specificity, positive predictive value (PPV), negative predictive value (NPV), and accuracy in the independent testing set, based on the case level*					
CNN model	96.7%	83.8%	90.0%	94.4%	91.5%
Senior radiologist	96.7	90.0	93.6	94.7	94.0
Junior radiologist	77.7	96.3	96.9	74.0	85.1

Sensitivity is TP/(TP + FN) × 100%. Specificity is TN/(TN + FP) × 100%. Positive predictive value (PPV) is TP/(TP + FP) × 100%. Negative predictive value (NPV) is TN/(TN + FN) × 100%. Accuracy is (TP + FN)/(TP + FN + TN + FN) × 100%

*CNN* convolutional neural network, *TP* true-positive, *FN* false-negative, *TN* true-negative, *FP* false-positive

## Discussion

In this study, we created a powerful CNN model for the detection of rib fractures by using chest radiographs. First, the quality of the input image was standardised. The CNN model was then trained to detect rib fractures with all lesions found. It showed promising results with high sensitivity and accuracy. Finally, a standardised model for rib fracture detection was developed. It outperformed the detection ability of senior and junior radiologists.

Deep learning has advanced significantly with new algorithms and optimised network structures, and these greatly contributed to the current study. Kim et al. [22] used X-ray-based AI to detect carpal fractures. The model showed sensitivity, specificity, and AUC values of 90%, 88% and 0.954, respectively [22]. Chung et al. [23] used a deep learning model to detect proximal humeral fractures, and the sensitivity, specificity, and AUC were 99%, 97% and 0.97, respectively [23]. AI showed promising results in fracture detection in the aforementioned two studies, as well as in this study. Three reasons could explain these results. The first reason is quality normalisation, as discussed in the preceding paragraph. The second reason is the application of the innovative network YOLOv3, which combines YOLOv2, Darknet-19 and other new residual networks. Compared to ResNet-152 and ResNet-101, YOLOv3 has better training speed and accuracy [37], further expanding its use. The third reason is that k-means was used to count the fracture marker box in the labelled sample. And result of the loss values of training and testing sets also proved the model performance. We could find the loss values of training and testing sets are hardly overlapping at the end of training. The reason is that the samples for training and testing sets were randomly obtained, and imbalance existed. While with the number of iterations increased, the curves tended to be more consistent, and the final training results showed the effectiveness of our detection algorithm.

A series of subgraphs was trained to locate multiple foci and were free of the hand-engineered region, which is rarely used in rib fracture detection. By examining three different scale feature maps, the number and specific locations of rib fractures could be better detected. Signal features detected by handcrafted analysis were challenged by the CNN model with a sliding window [38, 39]. Comparative testing showed that the sensitivity for detecting rib fractures was significantly higher with the CNN model than that by the junior radiologist and close to that by the senior radiologist at the fracture level. In addition, the precision of the model was slightly lower than that of the radiologists, although the model can still provide radiologists with specific locations for suspect fractures, thereby reducing the rate of lateral missed diagnosis.

The CNN is the most commonly used AI technique for medical imaging [17, 18]. The CNN model is also becoming a popular constituent of medical diagnosis with respect to efficiency and to precision medicine. Studies [3, 40] have shown that specific organ injuries are often correlated with a specific fractured rib [3, 40]. The number of displaced rib fractures could also be a strong predictor for developing pulmonary complications [41], which makes the detection of rib fractures important to prevent complications and help mitigate patient pain. This model used FROC to test its multilesion detection ability. The sensitivity was 91.3% when the false-positive rate of each case was set at 0.56. By comparison, only 49% of rib fractures are traditionally detected on the physical evaluation of radiographs [42]. This result may expand the clinical value of chest radiographs and reduce the rate of recommendations for additional imaging (RAIs). Harvey et al. [7] reported that the rate of RAIs have increased by as much as 200% since 1995. In particular [7], in radiographic imaging of the chest, the increase is because of the low diagnostic accuracy of radiography. Some critics have implicated RAIs as a cause of the increased use of additional imaging and associated costs.

The process of image standardisation also includes some image enhancement techniques so that images from different devices can have the same image quality. An important factor is that image standardisation forms the basis for the performance of the CNN model. Nearly all radiology applications are highly dependent on radiographic image quality, especially when combined with AI. However, image quality standardisation has long presented a challenge and has affected the intelligent diagnostic development of radiography, ultrasonography, CT, and magnetic resonance imaging. Several methods have been proposed to solve this problem. As in the research by Li et al. [43], several steps were performed for standardisation, including rescaling, downsizing, and transformation. Smoothing, normalisation, and resampling have also been performed in diabetic retinopathy research [44]. However, most studies have focused on noise elimination or uniform size rather than on feature enhancement. In the current study, proper image enhancement to reduce the variability of different machine images was necessary, particularly because images were reviewed by different display systems.

Among various imaging modalities, chest radiography is the appropriate initial imaging modality for patients with rib fractures. CT may provide a more accurate diagnosis; however, it is usually only performed after diagnostic chest radiography [8]. Missed diagnoses of rib fractures on chest radiographs may cause legal disputes, especially in traffic accidents and physical fights. It more importantly may lead to delayed treatment. Therefore, this study focused on chest radiography for the early detection of rib fractures. The developed CNN model has a wide range of real-life applications, including but not limited to the following four areas: (1) assisting clinicians in an initial diagnosis of images; (2) screening images for errors after clinicians have made a diagnosis; (3) detecting rib fractures in unlabelled images in subsequent research studies; and (4) serving other studies that have been linked to rib fractures.

### Limitations

This study has few limitations, despite its promising results. First, the fractures on the radiographs were labelled according to the physician’s comprehensive diagnosis without gold standard modalities such as pathology. Second, only posteroanterior radiographs were obtained, and the lateral position of the rib was not considered. Third, radiography cannot consistently demonstrate fractures in the costal cartilage, which is an inherent problem that decreases the detection rate. Fourth, the model was trained and tested by using different image resolutions. The off-label use in clinical environment of the model deserves further research. Finally, because only 19,974 subgraphs (918 radiographs) were included to train the model, more radiographs should be enrolled in the training data to improve model efficacy. This study is only a preliminary attempt at using a CNN model to examine rib fractures, based on radiographs. The efficiency of CNN data models is expected to continue to improve with the advent of computer technology and big data.

## Conclusions

In this study, we proposed a model for detecting multiple rib fractures, by using a CNN based on quality-normalised chest radiographs through data collection from multicentre, image quality normalisation, CNN model construction, the model’s performance validation and its comparison with radiologists. The CNN model showed high diagnostic efficiency, which indicated that CNN can improve the detection rate of rib fractures on chest radiographs, help reduce missed diagnoses, avoid medical accidents, and relieve radiologists’ workload. Implementing AI models in a clinic is the tendency of medical development. Our research implies the potential value of using CNN in rib fracture diagnoses. The detection ability requires further validation, although CNN is promising for medical diagnosis.

## Data Availability

The datasets used and/or analysed during the current study are available from the corresponding author on reasonable request.

## Abbreviations

AI: Artificial intelligence
AUC: Area under the curve
CI: Confidence intervals
CT: Computed tomography
DR: Digital radiography
FROC: Free-response receiver operating characteristic
MUSICA: Multi-scale image contrast amplification
RAIs: Recommendations for additional imaging
ROC: Receiver operating characteristic
YOLO: You Only Look Once

## References

[1] Battle C, Lovett S, Hutchings H, Evans PA (2014). Predicting outcomes after blunt chest wall trauma: development and external validation of a new prognostic model. Crit Care.

[2] Dogrul BN, Kiliccalan I, Asci ES, Peker SC (2020). Blunt trauma related chest wall and pulmonary injuries: an overview. Chin J Traumatol.

[3] Liman ST, Kuzucu A, Tastepe AI, Ulasan GN, Topcu S (2003). Chest injury due to blunt trauma. Eur J Cardiothorac Surg.

[4] Peek J, Ochen Y, Saillant N, Groenwold RHH, Leenen LPH, Uribe-Leitz T (2020). Traumatic rib fractures: a marker of severe injury. A nationwide study using the National Trauma Data Bank. Trauma Surg Acute Care Open.

[5] Ziegler DW, Agarwal NN (1994). The morbidity and mortality of rib fractures. J Trauma.

[6] Chien CY, Chen YH, Han ST, Blaney GN, Huang TS, Chen KF (2017). The number of displaced rib fractures is more predictive for complications in chest trauma patients. Scand J Trauma Resusc Emerg Med.

[7] Harvey HB, Gilman MD, Wu CC, Cushing MS, Halpern EF, Zhao J (2015). Diagnostic yield of recommendations for chest CT examination prompted by outpatient chest radiographic findings. Radiology.

[8] Henry TS, Kirsch J, Kanne JP, Chung JH, Donnelly EF, Ginsburg ME (2014). ACR Appropriateness Criteria® rib fractures. J Thorac Imaging.

[9] Siela D (2008). Chest radiograph evaluation and interpretation. AACN Adv Crit Care.

[10] Chung JH, Cox CW, Mohammed T-LH, Kirsch J, Brown K, Dyer DS (2014). ACR appropriateness criteria blunt chest trauma. J Am Coll Radiol.

[11] Davis S, Affatato A (2006). Blunt chest trauma: utility of radiological evaluation and effect on treatment patterns. Am J Emerg Med.

[12] Dubinsky I, Low A (1997). Non-life-threatening blunt chest trauma: appropriate investigation and treatment. Am J Emerg Med.

[13] Kahn CE (2017). From images to actions: opportunities for artificial intelligence in radiology. Radiology.

[14] Kermany DS, Goldbaum M, Cai W, Valentim CCS, Liang H, Baxter SL (2018). Identifying medical diagnoses and treatable diseases by image-based deep learning. Cell.

[15] Haenssle HA, Fink C, Schneiderbauer R, Toberer F, Buhl T, Blum A (2018). Man against machine: diagnostic performance of a deep learning convolutional neural network for dermoscopic melanoma recognition in comparison to 58 dermatologists. Ann Oncol.

[16] Yamashita R, Nishio M, Do RKG, Togashi K (2018). Convolutional neural networks: an overview and application in radiology. Insights Imaging.

[17] Wernick MN, Yang Y, Brankov JG, Yourganov G, Strother SC (2010). Machine learning in medical imaging. IEEE Signal Process Mag.

[18] Kohli M, Prevedello LM, Filice RW, Geis JR (2017). Implementing machine learning in radiology practice and research. Am J Roentgenol.

[19] Liang M, Tang W, Xu DM, Jirapatnakul AC, Reeves AP, Henschke CI (2016). Low-dose CT screening for lung cancer: computer-aided detection of missed lung cancers. Radiology.

[20] Lu F, Wu F, Hu P, Peng Z, Kong D (2017). Automatic 3D liver location and segmentation via convolutional neural network and graph cut. Int J Comput Assist Radiol Surg.

[21] Kooi T, Litjens G, van Ginneken B, Gubern-Mérida A, Sánchez CI, Mann R (2017). Large scale deep learning for computer aided detection of mammographic lesions. Med Image Anal.

[22] Kim DH, MacKinnon T (2018). Artificial intelligence in fracture detection: transfer learning from deep convolutional neural networks. Clin Radiol.

[23] Chung SW, Han SS, Lee JW, Oh KS, Kim NR, Yoon JP (2018). Automated detection and classification of the proximal humerus fracture by using deep learning algorithm. Acta Orthop.

[24] Olczak J, Fahlberg N, Maki A, Razavian AS, Jilert A, Stark A (2017). Artificial intelligence for analyzing orthopedic trauma radiographs: deep learning algorithms—Are they on par with humans for diagnosing fractures?. Acta Orthop.

[25] Bg A, Jy B, Sw A, Gz A, Yz A, Xw A, Mw A (2022). Automatic detection and localization of thighbone fractures in X-ray based on improved deep learning method. Comput Vis Image Underst.

[26] Jin L, Yang J, Kuang K, Ni B, Gao Y, Sun Y, Gao P, Ma W, Tan M, Kang H (2020). Deep-learning-assisted detection and segmentation of rib fractures from CT scans: development and validation of FracNet. EBioMedicine.

[27] Weikert T, Noordtzij LA, Bremerich J, Stieltjes B, Parmar V, Cyriac J, Sommer G, Sauter AW (2020). Assessment of a deep learning algorithm for the detection of rib fractures on whole-body trauma computed tomography. Korean J Radiol.

[28] Yang C, Wang J, Xu J, Huang C, Liu F, Sun W, Hong R, Zhang L, Ma D, Li Z (2022). Development and assessment of deep learning system for the location and classification of rib fractures via computed tomography. Eur J Radiol.

[29] Ren S, He K, Girshick R, Sun J (2015). Faster r-cnn: towards real-time object detection with region proposal networks. Adv Neural Inf Process Syst.

[30] Pang J, Chen K, Shi J, Feng H, Ouyang W, Lin D. Libra r-cnn: towards balanced learning for object detection. In: Proceedings of the IEEE/CVF conference on computer vision and pattern recognition; 2019; p. 821–30.

[31] Zhang H, Chang H, Ma B, Wang N, Chen X. Dynamic R-CNN: towards high quality object detection via dynamic training. In: Computer vision-ECCV; 2020. p. 12360.

[32] Cai Z, Vasconcelos N. Cascade R-CNN: delving into high quality object detection. In: IEEE/CVF conference on computer vision and pattern recognition; 2018. p. 6154–62.

[33] Gao Y, Liu H, Jiang L, Yang C, Yin X, Coatrieux J-L, Chen Y (2022). CCE-Net: a rib fracture diagnosis network based on contralateral, contextual, and edge enhanced modules. Biomed Signal Process Control.

[34] Redmon J, Farhadi A. Yolov3: an incremental improvement. 2018. arXiv preprint arXiv:1804.02767.

[35] Staege MS (2016). Gene expression music algorithm-based characterization of the Ewing sarcoma stem cell signature. Stem Cells Int.

[36] Sun M, Wang Y, le Bastard C, Pan J, Ding Y (2017). Signal subspace smoothing technique for time delay estimation using MUSIC algorithm. Sensors.

[37] Kim K-J, Kim P-K, Chung Y-S, Choi D-H. Performance enhancement of yolov3 by adding prediction layers with spatial pyramid pooling for vehicle detection. In: 2018 15th IEEE international conference on advanced video and signal based surveillance (AVSS); 2018. p. 1–6.

[38] Liao C, Bilgic B, Manhard MK, Zhao B, Cao X, Zhong J (2017). 3D MR fingerprinting with accelerated stack-of-spirals and hybrid sliding-window and GRAPPA reconstruction. Neuroimage.

[39] Tsui P-H, Chen CK, Kuo WH, Chang KJ, Fang J, Ma HY, Chou D (2017). Small-window parametric imaging based on information entropy for ultrasound tissue characterization. Sci Rep.

[40] Ivey KM, White CE, Wallum TE, Aden JK, Cannon JW, Chung KK (2012). Thoracic injuries in US combat casualties: a 10-year review of Operation Enduring Freedom and Iraqi Freedom. J Trauma Acute Care Surg.

[41] Talbot BS, Gange CP, Chaturvedi A, Klionsky N, Hobbs SK, Chaturvedi A (2017). Traumatic rib injury: patterns, imaging pitfalls, complications, and treatment. Radiographics.

[42] Crandall J, Kent R, Patrie J, Fertile J, Martin P. Rib fracture patterns and radiologic detection–a restraint-based comparison. In: Annual proceedings/association for the advancement of automotive medicine. Association for the Advancement of Automotive Medicine; 2000. p. 235. PMC3217385 11558086

[43] Li Z, Keel S, Liu C, He Y, Meng W, Scheetz J (2018). An automated grading system for detection of vision-threatening referable diabetic retinopathy on the basis of color fundus photographs. Diabet Care.

[44] Gulshan V, Peng L, Coram M, Stumpe MC, Wu D, Narayanaswamy A (2016). Development and validation of a deep learning algorithm for detection of diabetic retinopathy in retinal fundus photographs. JAMA.

